# Effects of Water-Retaining Agent Application on Growth Physiological Characteristics and Yield of Alfalfa (*Medicago sativa* L.)

**DOI:** 10.3390/plants15091304

**Published:** 2026-04-23

**Authors:** Minhua Yin, Mingzhu Wang, Wenqiong Ma, Yuanbo Jiang, Wenjing Chang, Yanxia Kang, Guangping Qi, Yanlin Ma, Guanheng Wu

**Affiliations:** 1 College of Water Conservancy and Hydropower Engineering, Gansu Agricultural University, Lanzhou 730070, China; yinmh@gsau.edu.cn (M.Y.); 1073324020379@st.gsau.edu.cn (M.W.); 16649875240@163.com (W.M.); 1073324120789@st.gsau.edu.cn (W.C.); qigp@gsau.edu.cn (G.Q.); mayl@gsau.edu.cn (Y.M.); 2 Irrigation Experiment Station of the Jingtai-Chuan Electric Irrigation Administration, Baiyin 730900, China; 18294900992@163.com

**Keywords:** water-retaining agent, growth, physiology, yield, principal component analysis, water use efficiency

## Abstract

In arid and semi-arid regions, the cultivation of artificial grasslands commonly suffers from low productivity due to insufficient water supply. The rational application of water-retaining agents is an important approach to alleviating production constraints in artificial grasslands facing resource-based water scarcity. This study investigated two types of water-retaining agents [starch-grafted acrylate water-retaining agent (B1) and polyacrylamide water-retaining agent (B2)] and four application rates [0 kg·hm^−2^ (CK), 30 kg·hm^−2^ (T1), 60 kg·hm^−2^ (T2), 90 kg·hm^−2^ (T3)], systematically analyzing their effects on the growth, osmotic adjustment substances, antioxidant enzyme activities, and yield of alfalfa. The results showed that alfalfa plant height, stem diameter, leaf area, branch number, soluble sugar (SS), soluble protein (SP), and proline (Pro) all exhibited a decreasing trend with increasing cutting times. The activities of superoxide dismutase (SOD), catalase (CAT), and peroxidase (POD) in alfalfa leaves initially increased and then decreased with increasing application rates of water-retaining agents, while malondialdehyde (MDA) content showed a decreasing trend. Under the B2T2 treatment, both alfalfa yield and water-use efficiency (WUE) reached their highest values, recorded as 4931.97 kg·hm^−2^ (2022), 6021.44 kg·hm^−2^ (2023) and 2.19 kg·m^−3^ (2022), 2.39 kg·m^−3^ (2023), respectively. Based on the principal component analysis for comprehensive evaluation, the B2T2 treatment (polyacrylamide water-retaining agent applied at 60 kg·hm^−2^) achieved the highest comprehensive score in both years and could synergistically improve alfalfa yield and water-use efficiency. However, its applicability in the Yellow River irrigation region of Gansu Province and similar ecological areas still requires further verification through field trials.

## 1. Introduction

Alfalfa (*Medicago sativa* L.) is a perennial leguminous forage species characterized by cold and drought tolerance, high yield, and excellent quality. It is primarily cultivated in regions such as Asia, Europe, and North America, making it the most widely planted artificial forage globally [1]. Furthermore, alfalfa features a lush canopy and a well-developed root system, and it can fix nitrogen through rhizobia, effectively enhancing ground cover, improving soil fertility, and mitigating soil erosion. This gives it a unique advantage in improving regional ecological environments [2]. To ensure national food security and alleviate the supply–demand contradiction between forage and livestock, China has successively formulated and implemented policies such as the “Action to Revitalize the Dairy Industry through Alfalfa Development”, the “National Plan for Grass-fed Livestock Development”, the “National Alfalfa Industry Development Plan”, and the “14th Five-Year Plan for National Forage Industry Development” [3]. According to statistics, in 2023, China’s alfalfa planting area and total output reached 551,000 hectares and 5 million tons, respectively. However, nearly a quarter of its supply still relies on imports, with a significant gap, particularly for high-quality alfalfa [4]. Alfalfa in China is mainly cultivated in arid and semi-arid regions of the northwest, including Inner Mongolia, Gansu, Ningxia, and Xinjiang, where water scarcity, poor soil, and extensive management practices result in low productivity [5]. Therefore, investigating water-saving and yield-increasing cultivation models for alfalfa under conditions of resource-based water scarcity is of great significance for promoting the sustainable and efficient development of the livestock industry.

Water is a key factor influencing the life activities of crops. Water deficit reduces the rate of biochemical reactions and decreases carbohydrate production, thereby limiting growth and development and constraining the productive potential of crops [6]. As a high-molecular-weight polymer, water-retaining agents exhibit outstanding water absorption, retention, and repeated absorption-release performance, forming a hydrogel upon swelling that gradually releases water. The application of water-retaining agents can create a micro-reservoir around the crop root zone, providing crops with a sustained water supply to a certain extent [7]. Studies have shown that the application of water-retaining agents increases oat yield by improving the number of panicles harvested, the number of grains per panicle, and the grain weight per panicle [8]. Potassium polyacrylate, fulvic acid, and polyacrylamide water-retaining agents can significantly enhance the leaf area index, chlorophyll content, net photosynthetic rate, and transpiration rate of maize during its early growth stages, with potassium polyacrylate exhibiting the most pronounced promotional effect [9]. The application of polyacrylate-type water-retaining agents has been shown to significantly promote crop growth; when applied to maize cultivated in the Hetao Plain, such agents enhanced soil fertility and markedly increased plant height and stem diameter [10]. The activities of superoxide dismutase, catalase, and peroxidase in wheat flag leaves first increased and then decreased with increasing application rates of attapulgite water-retaining agents, while malondialdehyde content in wheat flag leaves first decreased and then increased [11]. The combined application of sodium polyacrylate water-retaining agents and chemical fertilizers significantly increased soil water content and promoted deeper root growth in wheat [12]. Saloome et al. [13] investigated the effects of different irrigation management strategies and varying application rates of Aquasource superabsorbent (AS) on mint yield and water-use efficiency (WUE) at the Agricultural Engineering Research Institute (AERI) in Karaj, and found that the moderate use of AS (0.5 wt%) reduced water consumption while improving essential oil yield and WUE in mint cultivation. Zhou et al. [14] examined the effects of different water-retaining agent concentrations on oat forage yield at the Tenhe and Ewenki experimental stations in Hulunbuir, Inner Mongolia Autonomous Region, and found that oat hay yield reached its maximum when the water-retaining agent application rate was 75 kg·hm^−2^. Dong et al. [15] investigated the effects of a novel composite water-retaining agent on wheat seedlings under drought stress at the experimental base of Qingdao Agricultural University, and found that under mild drought stress conditions, a novel composite water-retaining agent composed of 5% rare earth, 55% attapulgite clay, 10% biochar, and 30% bentonite increased the activities of antioxidant enzymes, the accumulation of osmotic adjustment substances, and antioxidant content in wheat leaves, thereby extending the growth cycle of wheat. Du et al. [16] demonstrated through superabsorbent polymer experiments that the application of superabsorbent polymers significantly increased wheat yield, thousand-grain weight, and the number of grains per spike. However, existing research has largely focused on the effects of water-retaining agents on the agronomic traits and yield of cereal crops such as maize and wheat, and has predominantly examined either a single type of water-retaining agent or a single application rate. Studies addressing the effects of different types and application rates of water-retaining agents on leguminous forages—particularly alfalfa—remain comparatively limited. Furthermore, systematic investigations into the intrinsic linkages among alfalfa growth dynamics, physiological and biochemical responses (such as osmotic adjustment substance accumulation and changes in antioxidant enzyme activity), and ultimate yield formation under water-retaining agent application are still lacking.

Gansu Province represents the largest alfalfa-growing region in China, with a cultivated area reaching 193,300 hm^2^ in 2023 [17]. As the alfalfa industry in Gansu has continued to develop in recent years, a series of challenges have become increasingly prominent, including limited regional natural precipitation, increasingly stringent controls on irrigation water use, rising costs of agricultural inputs and land rental, and strict policies governing the use of cultivated land. The problem of high costs and low returns in alfalfa production is therefore in urgent need of resolution [18]. We hypothesized that applying an appropriate type and rate of water-retaining agent can effectively reduce ineffective soil evaporation, enhance soil water availability, and strengthen leaf antioxidant defense capacity and osmotic adjustment ability, thereby promoting crop growth and improving water-use efficiency. In light of this, the present study takes alfalfa cultivated in the Yellow River irrigation district of Gansu Province as the research subject, with the following objectives: (1) To analyze the effects of water-retaining agent type and application rate on alfalfa growth, physiology, and yield; (2) to elucidate the relationships among alfalfa growth, osmotic adjustment substances, antioxidant enzyme activity, and productive performance under WRA application; and (3) to employ methods such as principal component analysis and comprehensive evaluation to identify suitable water-retaining agent application regimes for alfalfa, with the aim of providing a theoretical basis for enhancing alfalfa productivity and promoting the healthy and sustainable development of the alfalfa industry in arid and semi-arid regions and ecologically similar areas.

## 2. Results

### 2.1. Effects of Water-Retaining Agent Application on the Growth Characteristics of Alfalfa

#### 2.1.1. Plant Height

**Figure 1:**
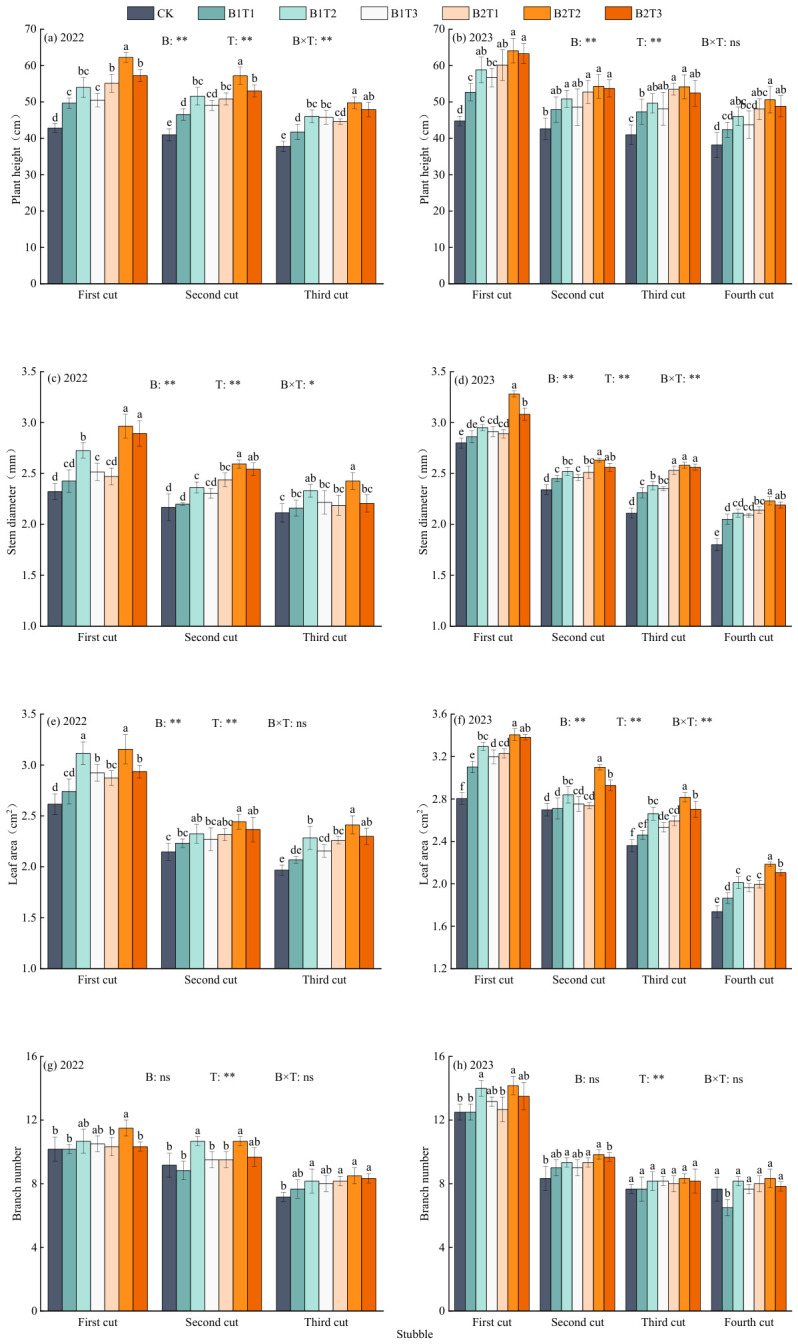
Effect of water-retaining agent on growth index of alfalfa. CK is the control; B1 is a starch grafting acrylate water-retaining agent; B2 is a polyacrylamide water-retaining agent. The application levels of T1 to T3 were 30 kg·hm^−2^ (T1), 60 kg·hm^−2^ (T2), and 90 kg·hm^−2^ (T3), respectively. Figures (**a**–**h**) represent the following: (**a**) plant height in 2022; (**b**) plant height in 2023; (**c**) stem diameter in 2022; (**d**) stem diameter in 2023; (**e**) leaf area in 2022; (**f**) leaf area in 2023; (**g**) branch number in 2022; (**h**) branch number in 2023. Different lowercase letters indicated a significant difference among treatments (*p* < 0.05). ** indicates a highly significant difference (*p* < 0.01); * indicates significant difference (*p* < 0.05); ns indicates that the difference is not significant (*p* > 0.05). The same below. Different lowercase letters indicate significant differences among treatments (Duncan’s multiple range test, *p* < 0.05).

Alfalfa plant height showed a decreasing trend with the progression of cutting cycles, and was highly significantly influenced by both water-retaining agent type and application rate, while their interaction effect was only extremely significant in 2022 (*p* < 0.01, Figure 1). Under the same application type, alfalfa plant height initially increased and then decreased with increasing water-retaining agent application rate, reaching its maximum at the T2 level. Under the same application level, alfalfa plant height followed the order B2 > B1. Among all treatments, alfalfa plant height was highest in the B2T2 treatment, exceeding that of other treatments by 11.62–39.27% in 2022 and 2.26~33.40% in 2023.

#### 2.1.2. Stem Diameter

Alfalfa stem diameter showed a decreasing trend with increasing cutting number and was significantly influenced by the type of water-retaining agent, application rate, and their interaction (*p* < 0.05, Figure 1). Under the same application type, alfalfa stem diameter first increased and then decreased with increasing application rate, following the order T2 > T3 > T1. Under the same application level, alfalfa stem diameter generally followed the order B2 > B1. Among all treatments, the maximum alfalfa stem diameter in both 2022 and 2023 occurred in the B2T2 treatment, which was 4.49~20.90% and 3.21~18.50% higher than the other treatments, respectively.

#### 2.1.3. Leaf Area

Alfalfa leaf area showed a gradually decreasing trend with the progression of cutting cycles and was significantly influenced by both water-retaining agent type and application rate, while their interaction effect was only extremely significant for alfalfa leaf area in 2023 (*p* < 0.01, Figure 1). Under the same application type, alfalfa leaf area in both 2022 and 2023 followed the order T2 > T3 > T1. Under the same application level, alfalfa leaf area in both 2022 and 2023 followed the order B2 > B1. Among all treatments, the B2T2 treatment achieved the maximum alfalfa leaf area, while the CK treatment yielded the minimum.

#### 2.1.4. Branch Number

Alfalfa branch number showed a gradually decreasing trend with the progression of cutting cycles, and was significantly influenced by application rate (*p* < 0.05, Figure 1g), while the effects of water-retaining agent type and their interaction were not significant. Under the same application type, alfalfa branch number in both 2022 and 2023 first increased and then decreased or remained unchanged with increasing application rate, with T2 being 10.06% and 6.80%, and 9.05% and 4.10% higher than T1 and T3 levels, respectively. Under the same application level, alfalfa branch number in both 2022 and 2023 followed the order B2 > B1. Across different treatments, the maximum alfalfa branch number in both 2022 and 2023 occurred under the B2T2 treatment (10.22 and 10.17), while the minimum was recorded under CK (8.83 and 9.04).

### 2.2. Effects of Water-Retaining Agent Application on the Leaf Physiological Characteristics of Alfalfa

#### 2.2.1. Osmotic Adjustment Substances

(1) Soluble protein content

**Figure 2:**
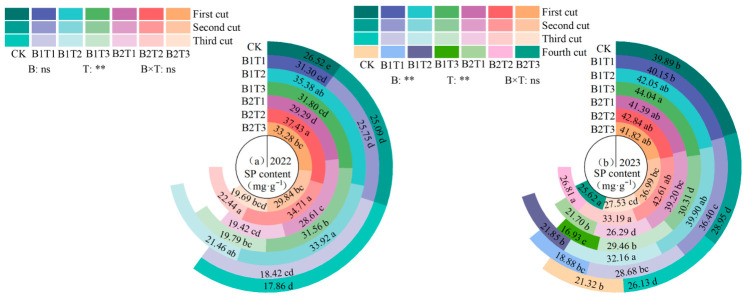
Effect of applying water-retaining agents on the soluble protein content in alfalfa leaves. (**a**) represents soluble protein content in 2022; (**b**) represents soluble protein content in 2023. Different lowercase letters indicate significant differences among treatments (Duncan’s multiple range test, *p* < 0.05).** indicates a highly significant difference (*p* < 0.01); ns indicates that the difference is not significant (*p* > 0.05).

Soluble protein (SP) content in alfalfa leaves showed a gradually decreasing trend with the progression of cutting cycles, and was highly significantly influenced by water-retaining agent application rate (*p* < 0.01, Figure 2). Water-retaining agent type was only extremely significant in 2023, while the interaction effect between the two factors was not significant (*p* > 0.05). Compared with CK, the increase in SP content across all treatments ranged from 7.38% to 34.57% in 2022 and from 3.82% to 25.07% in 2023, with the B2T2 treatment showing the largest increase (36.14% and 25.08%, respectively). Under the same application type, the T2 level was generally optimal overall. Under the B1 application type, the order was T2 > T3 > T1 in 2022 and T2 > T1 > T3 in 2023; under the B2 application type, the order was T2 > T3 > T1 in both years. Under the same application level, leaf SP content at the T2 level was significantly higher than that at the T1 and T3 levels. Among all treatments, the B2T2 treatment yielded the highest SP content, which was 36.14% and 25.08% higher than the CK treatment in 2022 and 2023, respectively.

(2) Soluble sugar content

**Figure 3:**
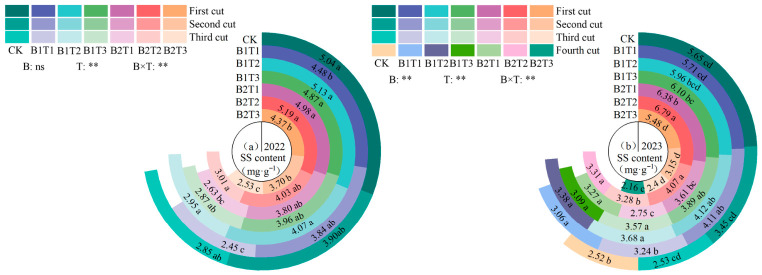
Effect of applying water-retaining agents on the soluble sugar content in alfalfa leaves. (**a**) represents soluble sugar content in 2022; (**b**) represents soluble sugar content in 2023. Different lowercase letters indicate significant differences among treatments (Duncan’s multiple range test, *p* < 0.05). ** indicates a highly significant difference (*p* < 0.01); ns indicates that the difference is not significant (*p* > 0.05).

Soluble sugar (SS) content in alfalfa leaves showed a decreasing trend with the progression of cutting cycles, and was highly significantly influenced by water-retaining agent application rate and the interaction effect of the two factors, while water-retaining agent type was only highly significant in 2023 (*p* < 0.01, Figure 3). Under the B1 treatment, leaf SS content followed the order T2 > T3 > T1 in both years. Under the B2 application type, leaf SS content followed the order T2 > T1 > T3 in both years. Under the T1 and T3 application levels, leaf SS content followed the order B1 > B2 (with the exception of the T1 application level in 2022). Under the T2 application level, leaf SS content followed the order B2 > B1. Among all treatments, the leaf SS content was highest in the B2T2 treatment, at 4.08 mg·g^−1^ (2022) and 4.36 mg·g^−1^ (2023), representing increases of 3.82% (2022) and 23.16% (2023) compared to the CK treatment.

(3) Proline content

**Figure 4:**
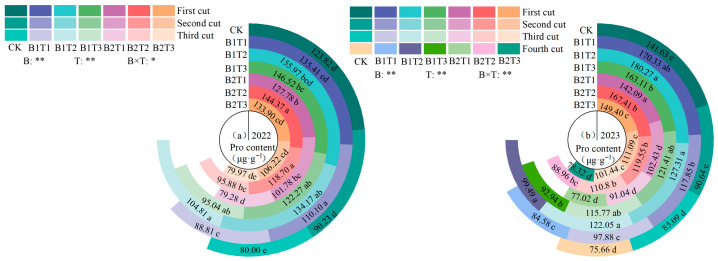
Effect of applying water-retaining agents on the proline content in alfalfa leaves. (**a**) represents proline content in 2022; (**b**) represents proline content in 2023. Different lowercase letters indicate significant differences among treatments (Duncan’s multiple range test, *p* < 0.05). ** indicates a highly significant difference (*p* < 0.01); * indicates significant difference (*p* < 0.05).

Proline (Pro) content in alfalfa leaves showed an increasing trend with the progression of planting years and was significantly influenced by the type of water-retaining agent, the application rate, and their interaction (*p* < 0.05, Figure 4). Under the same application type, leaf Pro content first increased and then decreased with increasing water-retaining agent application rate, reaching its maximum at the T2 level, with values of 376.96 μg·g^−1^ in 2022 and 507.93 μg·g^−1^ in 2023, respectively. Under the same application level, leaf Pro content consistently followed the order B1 > B2. Among all treatments, the B1T2 treatment yielded the highest leaf Pro content in both 2022 and 2023, exceeding that of other treatments by 8.56~34.32% in 2022 and 7.28~34.63% in 2023, respectively.

#### 2.2.2. Antioxidant System

(1) Superoxide dismutase activity

**Figure 5:**
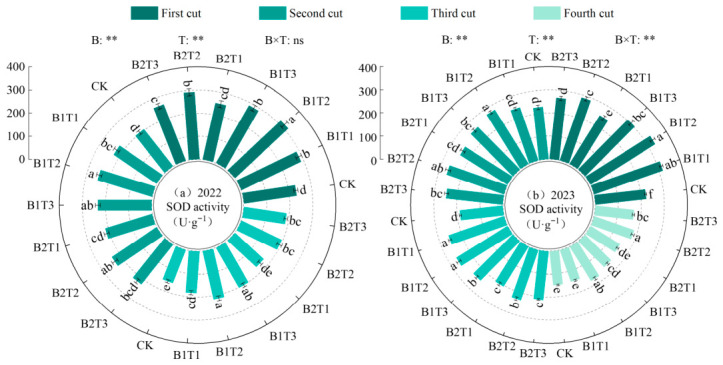
Effect of applying water-retaining agents on the superoxide dismutase activity in alfalfa leaves. (**a**) represents Superoxide dismutase activity in 2022; (**b**) represents Superoxide dismutase activity in 2023. Different lowercase letters indicate significant differences among treatments (Duncan’s multiple range test, *p* < 0.05). ** indicates a highly significant difference (*p* < 0.01); ns indicates that the difference is not significant (*p* > 0.05).

Superoxide dismutase (SOD) activity in alfalfa leaves showed an increasing trend with the progression of planting years and was highly significantly influenced by both water-retaining agent type and application rate, while their interaction effect was only highly significant in 2023 (*p* < 0.01, Figure 5). Under the same application type, leaf SOD activity first increased and then decreased with increasing water-retaining agent application rate, reaching a maximum at the T2 level. Under the same application level, leaf SOD activity followed the order B1 > B2. Among all treatments, leaf SOD activity reached its maximum under the B1T2 treatment, which was 36.17% and 33.12% higher than CK in 2022 and 2023, respectively.

(2) Peroxidase activity

**Figure 6:**
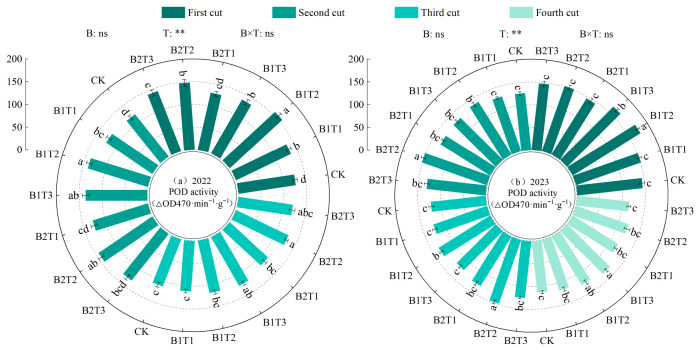
Effect of applying water-retaining agents on the peroxidase activity in alfalfa leaves. (**a**) represents Peroxidase activity in 2022; (**b**) represents Peroxidase activity in 2023. Different lowercase letters indicate significant differences among treatments (Duncan’s multiple range test, *p* < 0.05). ** indicates a highly significant difference (*p* < 0.01); ns indicates that the difference is not significant (*p* > 0.05).

Peroxidase (POD) activity in alfalfa leaves was highly significantly influenced by water-retaining agent application rate, while the effects of water-retaining agent type and their interaction were not significant (*p* > 0.05, Figure 6). Under the same application type, leaf POD activity first increased and then decreased with increasing water-retaining agent application rate, following the order T2 > T3 > T1. Under the same application level, the difference between types was opposite between years: B2 was higher than B1 by 0.45% in 2022, while B1 was higher than B2 by 1.94% in 2023. Among all treatments, the maximum leaf POD activity occurred under the B2T2 treatment, reaching 139.62 ΔOD470·min^−1^·g^−1^ in 2022 and 143.16 ΔOD470·min^−1^·g^−1^ in 2023, which were 1.39~17.42% and 0.27~13.38% higher than the other treatments, respectively.

(3) Catalase activity

**Figure 7:**
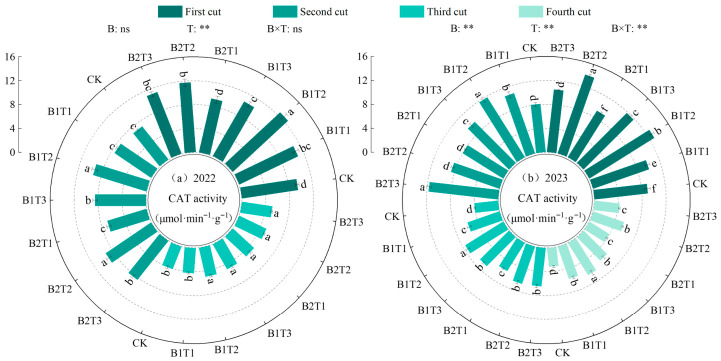
Effect of applying water-retaining agents on the catalase activity in alfalfa leaves. (**a**) represents Catalase activity in 2022; (**b**) represents Catalase activity in 2023. Different lowercase letters indicate significant differences among treatments (Duncan’s multiple range test, *p* < 0.05). ** indicates a highly significant difference (*p* < 0.01); ns indicates that the difference is not significant (*p* > 0.05).

Catalase (CAT) activity in alfalfa leaves was highly significantly influenced by water-retaining agent application rate, while the effects of water-retaining agent type and their interaction were only highly significant in 2023 (*p* < 0.01, Figure 7). Under the same application type, leaf CAT activity first increased and then decreased with increasing water-retaining agent application rate, consistently following the order T2 > T3 > T1. Under the same application level, the CAT activity followed the order B1 > B2. Among all treatments, the maximum leaf CAT activity occurred under the B1T2 treatment in both years, with values of 8.94 μmol·min^−1^·g^−1^ (2022) and 9.59 μmol·min^−1^·g^−1^ (2023), representing increases of 3.55~30.51% (2022) and 11.06~58.46% (2023) compared to other treatments.

(4) Malondialdehyde content

**Figure 8:**
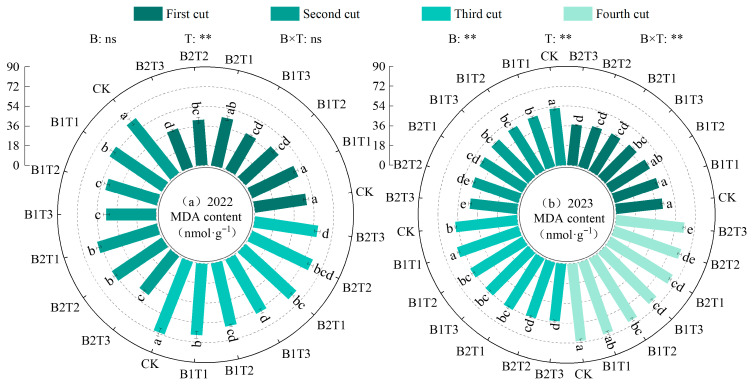
Effect of applying water-retaining agents on the malondialdehyde content in alfalfa leaves. (**a**) represents malondialdehyde content in 2022; (**b**) represents malondialdehyde content in 2023. Different lowercase letters indicate significant differences among treatments (Duncan’s multiple range test, *p* < 0.05). ** indicates a highly significant difference (*p* < 0.01); ns indicates that the difference is not significant (*p* > 0.05).

Malondialdehyde (MDA) content in alfalfa leaves increased with the progression of cutting cycles and was highly significantly influenced by water-retaining agent application rate, while the effects of water-retaining agent type and their interaction were only highly significant in 2023 (*p* < 0.01, Figure 8). Under the same application type, leaf MDA content showed a decreasing trend with increasing water-retaining agent application rate, consistently following the order T1 > T2 > T3. Under the same application level, the difference between types was opposite between years: B2 was higher than B1 by 1.12% in 2022, while B1 was higher than B2 by 6.24% in 2023. Among all treatments, the CK treatment yielded the highest MDA content, which was 7.78~29.94% and 1.06~10.54% higher than the other treatments in 2022 and 2023, respectively.

### 2.3. Effects of Water-Retaining Agent Application on Alfalfa Yield

**Figure 9:**
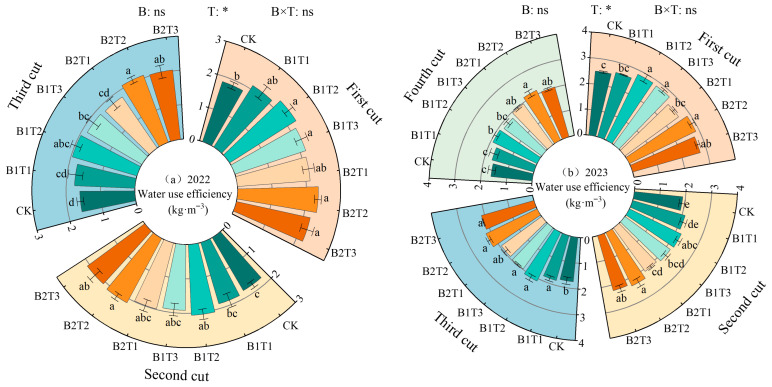
Effect of water-retaining agents application on alfalfa yield. (**a**) represents yield in 2022; (**b**) represents yield in 2023. Different lowercase letters indicate significant differences among treatments (Duncan’s multiple range test, *p* < 0.05). ** indicates a highly significant difference (*p* < 0.01); * indicates significant difference (*p* < 0.05); ns indicates that the difference is not significant (*p* > 0.05).

Alfalfa yield showed a decreasing trend with the progression of cutting cycles, and was significantly influenced by water-retaining agent type, application rate, and their interaction effect (*p* < 0.05, Figure 9). Under the same water-retaining agent type, alfalfa yield first increased and then decreased with increasing application rate, following the order T2 > T3 > T1. Under the same application level, alfalfa yield consistently followed the order B2 > B1. Over the two years, the application of water-retaining agents significantly increased alfalfa yield, with the B2T2 treatment achieving the maximum values of 4931.97 kg·hm^−2^ in 2022 and 6021.44 kg·hm^−2^ in 2023. Alfalfa yield under water-retaining agent application was 18.75% and 8.66% higher than that without water-retaining agent application in 2022 and 2023, respectively.

### 2.4. Effects of Water-Retaining Agent Application on Alfalfa Water-Use Efficiency

**Figure 10:**
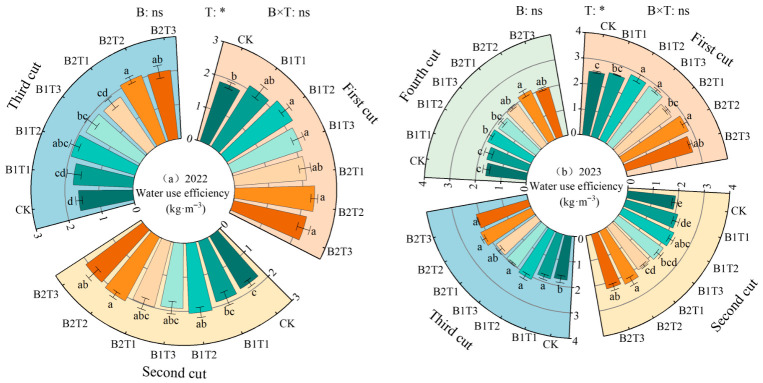
Effect of water-retaining agents on water-use efficiency in alfalfa. (**a**) represents water use efficiency in 2022; (**b**) represents water use efficiency in 2023. Different lowercase letters indicate significant differences among treatments (Duncan’s multiple range test, *p* < 0.05). * indicates significant difference (*p* < 0.05); ns indicates that the difference is not significant (*p* > 0.05).

Alfalfa water-use efficiency (WUE) decreased with the progression of cutting cycles and was significantly influenced by water-retaining agent application rate; water-retaining agent type was only significant in 2023, while the interaction effect of the two factors was not significant (*p* > 0.05, Figure 10). Compared with CK, alfalfa WUE under water-retaining agent application increased by 9.70~25.46% in 2022 and 6.32~23.52% in 2023. Under the same application type, alfalfa WUE followed the order T2 > T3 > T1. Under the same application level, alfalfa WUE followed the order B2 > B1. Among all treatments, the maximum alfalfa WUE occurred under the B2T2 treatment in both years, with values of 2.19 kg·m^−3^ in 2022 and 2.39 kg·m^−3^ in 2023.

### 2.5. Correlation Analysis Between Alfalfa Yield and Growth and Physiological Indicators

**Figure 11:**
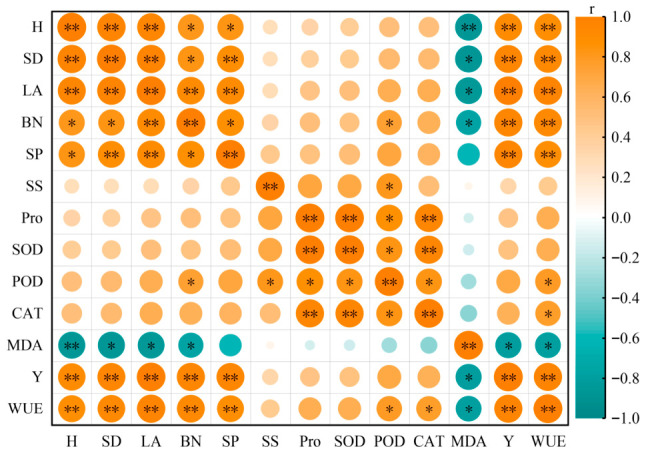
Correlation analysis between yield and growth physiological indexes. H, SD, LA, BN, Y, and WUE represent plant height, stem diameter, leaf area, branch number, yield, and water-use efficiency, respectively. ** indicates a highly significant difference (*p* < 0.01); * indicates significant difference (*p* < 0.05).

A correlation analysis was performed on alfalfa growth indicators, leaf osmotic adjustment substances, leaf antioxidant system parameters, yield, and water-use efficiency under different water-retaining agent treatments (Figure 11). The results indicated that yield was significantly positively correlated with the growth indicators plant height, stem diameter, leaf area, and branch number, as well as with SP content, SS content, Pro content, SOD activity, POD activity, CAT activity, and water-use efficiency, while it was highly significantly negatively correlated with MDA content.

### 2.6. Principal Component Analysis of Alfalfa Yield and Growth and Physiological Indicators

**Figure 12:**
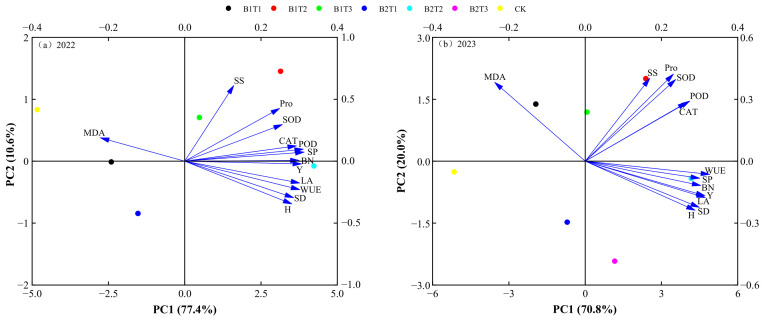
Principal component analysis of alfalfa yield and growth–physiological indicators. H, SD, LA, BN, Y, and WUE represent plant height, stem diameter, leaf area, branch number, yield, and water-use efficiency. (**a**) PCA of yield and growth-physiological indicators for 2022; (**b**) PCA of yield and growth-physiological indicators for 2023.

**Figure 13:**
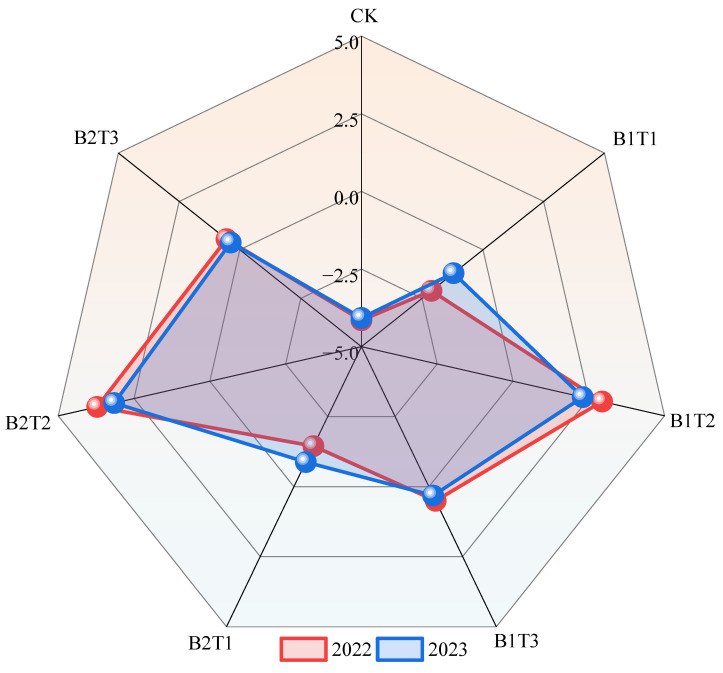
Principal component scores for each treatment.

Principal component analysis (Figure 12) indicated that in 2022, the variance contribution rates of PC1 and PC2 were 77.4% and 10.6%, respectively, with a cumulative variance of 88.0%. In 2023, the variance contribution rates of PC1 and PC2 were 70.8% and 20.0%, respectively, with a cumulative variance of 90.8%. Meanwhile, growth indicators (plant height, stem diameter, leaf area, branch number, and yield), antioxidant parameters (SOD, POD, and CAT), and osmotic adjustment substances (SS, SP, and Pro) were all significantly positively correlated with one another across both years, while malondialdehyde (MDA) was significantly negatively correlated with the other indicators. Based on the comprehensive scores of each treatment (Figure 13), among all treatments across the two years, the B2T2 treatment received the highest score, preliminarily indicating that, under the conditions of this experiment, the B2T2 treatment is a suitable water-retaining agent application mode for alfalfa cultivation.

## 3. Discussion

### 3.1. Effects of Water-Retaining Agent Application on the Growth Characteristics of Alfalfa

The application of water-retaining agents can enhance soil water retention capacity, improve the soil microenvironment for crop root growth, and thereby promote crop growth and development. Xu et al. [19] conducted research in Shandong Province and found that the application of water-retaining agents increased plant height, spike length, and the length of the internode below the spike of winter wheat by 1.6~9.8 cm, 0.09~0.57 cm, and 0.2~1.4 cm, respectively. Wang et al. [20] conducted research in the Guanzhong region of Shaanxi Province and found that the application of water-retaining agents significantly increased the seedling emergence rate (24.71%), maximum tiller number (115.71%), and spike number (72.78%) of winter wheat compared with no water-retaining agent application. Li et al. [21] conducted research in the Ningxia Hui Autonomous Region and found that under straw incorporation conditions, maize plant height and stem diameter during the growing period first increased and then decreased with increasing water-retaining agent application rate, with better maize growth performance observed at application rates of 60~90 kg·hm^−2^. In the present study, alfalfa plant height, stem diameter, leaf area, and branch number were significantly influenced by water-retaining agent application rate (*p* < 0.05). Furthermore, alfalfa plant height, stem diameter, leaf area, and branch number all first increased and then decreased with increasing water-retaining agent application rate, reaching their maximum values under the polyacrylamide water-retaining agent treatment at 60 kg·hm^−2^. This may be attributable to the fact that excessive water-retaining agent application reduces total soil porosity and increases soil bulk density, leading to soil hardening; only an appropriate amount of water-retaining agent can improve soil aggregate structure, enhance water-use efficiency, and provide more favorable growing conditions for crops [22]. In addition, other studies have shown [23] that polyacrylamide water-retaining agents promote alfalfa growth more effectively than starch-grafted acrylate water-retaining agents, which is consistent with the findings of the present study. This may be attributable to the improvement of soil moisture status in the root zone by the water-retaining agent, which provided the necessary turgor pressure basis for cell elongation. According to acid theory, sufficient water supply helps maintain cell turgor pressure, thereby promoting the elongation growth of alfalfa stems and leaves [24].

### 3.2. Effects of Water-Retaining Agent Application on the Leaf Physiological Characteristics of Alfalfa

Osmotic adjustment is central to maintaining cellular water balance in plants. The driving force for plant water uptake originates from the water potential gradient between the soil and the plant; when soil water potential is higher than root cell water potential, water enters the plant along this gradient [25]. Under mild water deficit conditions, plants actively accumulate organic osmotic regulatory substances such as soluble sugars, soluble proteins, and proline to lower cell osmotic potential and consequently reduce leaf water potential, thereby maintaining a water potential gradient favorable for water uptake from the soil and ensuring normal cellular physiological metabolism [26]. This study found that the water-retaining agent application rate significantly increased the contents of soluble protein, soluble sugar, and proline. This may be attributed to the fact that although the application of water-retaining agents improved soil water supply in the root zone and raised soil water potential, it also simultaneously promoted the synthesis and accumulation of osmotic regulatory substances, which correspondingly reduced cell water potential. As a result, a water potential gradient favorable for sustained water uptake was re-established at a higher soil water potential level [27]. In addition, the present study found that soluble protein content, soluble sugar content, and proline content all first increased and then decreased with increasing water-retaining agent application rate, reaching their maximum values at the 60 kg·hm^−2^ application level. This is inconsistent with the finding of Dai et al. [28] that soluble sugar content showed little variation across different treatments. A possible reason is that the microbial water-retaining agent (B3) used in the study by Dai et al. may have altered plant carbon allocation through microbial activity [29], such that soluble sugar did not accumulate as the primary responsive substance, while proline became a more sensitive osmotic adjustment indicator, thereby giving rise to the observed differences in the pattern of variation in soluble sugar content.

POD, SOD, and CAT are the primary antioxidant defense enzymes in plants, functioning to scavenge reactive oxygen species generated by environmental stress. Through mutual synergy, they maintain stable free radical levels, prevent cellular damage, and ensure normal physiological metabolism in plants. Studies have shown that the application of water-retaining agents under drought stress conditions significantly increases SOD, POD, and CAT activities in the leaves of Sophora davidii seedlings [30]. In the present study, SOD, POD, and CAT activities were highly significantly influenced by water-retaining agent application rate (*p* < 0.01). Furthermore, the present study found that SOD, POD, and CAT activities in alfalfa leaves under water-retaining agent application were significantly higher than those without water-retaining agent application by 7.66~33.12%, 2.57~13.39%, and 9.75~58.51%, respectively. This may be attributable to the fact that the application of water-retaining agents promotes plant absorption of water and nutrients, enhances the capacity for photosynthesis, maintains energy metabolic balance, reduces reactive oxygen species generation, and thereby elevates plant antioxidant metabolic levels [31]. Zhou et al. [32] conducted research in Hunan Province and found that the application of water-retaining agents under drought stress significantly reduced MDA content in Euonymus fortunei leaves, with MDA content showing an increasing trend with prolonged stress duration but a continuously decreasing trend with increasing water-retaining agent application rate. The present study also found that the application of both starch-grafted acrylate and polyacrylamide water-retaining agents reduced MDA content in alfalfa leaves, indicating that water-retaining agent application can alleviate damage to crop membrane structures and mitigate oxidative damage in plants under current water conditions [33].

### 3.3. Effects of Water-Retaining Agent Application on Alfalfa Yield and Water Use

The application of an appropriate amount of water-retaining agent can improve soil moisture conditions, reduce evaporation, increase soil water-holding capacity, provide a favorable growing environment for crops, and thereby enhance crop yield to a certain extent. Tian et al. [34] conducted research in the Inner Mongolia Autonomous Region and found that at oat row spacings of 20 cm, 15 cm, and 10 cm, the application of water-retaining agents significantly increased oat grain yield and aboveground biomass. Liu et al. [35] conducted research in Shanxi Province and found that the application of potassium polyacrylate, fulvic acid, and polyacrylamide water-retaining agents all significantly increased maize yield by 21.56%, 16.81%, and 11.83%, respectively, compared with no water-retaining agent application. This is similar to the finding of the present study that alfalfa yield under water-retaining agent application was 2.98~12.10 and 7.28~19.26 g·pot^−1^ higher than that without water-retaining agent application. Jing et al. [36] reported that alfalfa individual plant dry weight was highly significantly positively correlated with individual plant leaf dry weight (*p* < 0.01) and significantly positively correlated with individual plant leaf number and transpiration rate (*p* < 0.05). The present study also found that yield was highly significantly positively correlated with the growth indicators plant height, stem diameter, leaf area, and branch number (*p* < 0.01), and significantly positively correlated with the physiological indicators SP content, POD activity, and CAT activity (*p* < 0.05), while significantly negatively correlated with the physiological indicator MDA content (*p* < 0.05). Furthermore, the principal component analysis conducted in the present study revealed that growth indicators (plant height, stem diameter, leaf area, branch number, and yield), antioxidant parameters (SOD, POD, and CAT), and osmotic adjustment substances (SS, SP, and Pro) were all significantly positively correlated with one another across both years, while malondialdehyde (MDA) was significantly negatively correlated with the other indicators. This comprehensively reflects the overall coordination between growth and the physiological status of alfalfa under appropriate water-retaining agent treatments. The decrease in MDA content may be associated with the improvement of water supply by the water-retaining agent and the alleviation of cell membrane lipid peroxidation damage [37].

The application of an appropriate amount of water-retaining agent is conducive to improving crop WUE. The present study found that the application of both B1 and B2 types increased alfalfa WUE by 0.17~0.36 kg·m^−3^ in 2022 and 0.12~0.34 kg·m^−3^ in 2023, and by 0.23~0.44 kg·m^−3^ in 2022 and 0.21~0.45 kg·m^−3^ in 2023, respectively, compared with no water-retaining agent application. A possible explanation is that water-retaining agents can store large amounts of water, and after incorporation into the soil, can slow the rate of soil water infiltration and reduce crop water consumption, thereby promoting crop growth and development and ultimately enhancing crop WUE [38]. Kang et al. [39] conducted long-term field experiments at the Henan Academy of Agricultural Sciences and found that wheat yield, ET, and WUE all showed an increasing trend with an increasing water-retaining agent application rate, reaching maximum values at an application rate of 45 kg·hm^−2^. In the present study, alfalfa WUE first increased and then decreased with increasing water-retaining agent application rate, reaching its maximum value (2.24 kg·m^−3^) at an application rate of 60 kg·hm^−2^. This differs from the above-mentioned conclusion, which may be related to the differences in the application levels set in the respective studies (0, 15, 30, and 45 kg·hm^−2^ in Kang et al.’s study versus 0, 30, 60, and 90 kg·hm^−2^ in the present study). The application rates and gradients in Kang et al.’s experiment were relatively small and had not yet reached the threshold for wheat growth and development, whereas the application rates and gradients in the present experiment were larger and had already reached the threshold for alfalfa growth and development. In addition, the present study found that alfalfa WUE showed a decreasing trend with the progression of cutting cycles. This may be because WUE is jointly determined by yield and water consumption; as yield decreases with increasing cutting number, and as the later cuttings of alfalfa mainly grow during summer and autumn when average temperatures are higher, leading to relatively greater crop water consumption, WUE consequently declines [40].

Based on a two-year pot experiment, the present study preliminarily clarified the regulatory effects of polyacrylamide water-retaining agent application on alfalfa growth and water use. The polyacrylamide water-retaining agent, owing to the presence of abundant hydrophilic groups in its three-dimensional network structure, can absorb large amounts of water and swell to form hydrogels after being incorporated into the soil. This slows down the infiltration rate of soil water and reduces surface evaporation losses, thereby improving soil water-holding capacity [41]. When crops require water, it supplies water continuously to the root zone in a slow-release manner, maintaining a relatively stable soil water potential in the root zone. This helps reduce stomatal conductance and transpiration rate, such that the decline in photosynthetic rate is smaller than that in transpiration rate, ultimately leading to improved WUE [42]. However, since the pot environment differs fundamentally from field production in terms of water movement, root growth space, and soil physicochemical processes, the applicability of the above results to field conditions still requires further verification. Future studies should combine field experiments to investigate the actual effects and stability of water-retaining agents under natural precipitation and complex soil conditions. In addition, nutrient management factors should be incorporated to explore the synergistic effects of combined application of water-retaining agents and fertilizers, systematically revealing the regulatory mechanisms of water-fertilizer and water-retaining agent coupling on alfalfa yield, quality, and soil microenvironment, with the aim of providing a more scientific theoretical basis for green and efficient alfalfa production in arid and semi-arid regions.

## 4. Materials and Methods

### 4.1. Description of the Experimental Site

The experiment was conducted from March to October during 2022–2023 at the Forage Practical Training Base of Gansu Agricultural University (36°09′ N, 103°69′ E, mean elevation of 1525 m). The experimental area is located in the transition zone between the Loess Plateau and the Qinghai–Tibet Plateau, and is characterized by a temperate semi-arid climate with dry conditions, abundant sunlight, relatively deep soil profiles, and fertile soils. The mean annual precipitation within the experimental area ranges from 400 to 600 mm, with an evaporation of 1410 mm, a mean annual temperature of 10.3 °C, a frost-free period of 180 days, and an annual sunshine duration of 2100–2600 h. The soil type is loessial soil (Huangmian soil), with a bulk density of 0.92 g·cm^−3^, a field water-holding capacity of 27.3% (volumetric water content), and a pH of 7.5. The baseline soil nutrient contents in the 0-30 cm soil layer were 68.14 mg·kg^−1^ for alkali-hydrolyzable nitrogen, 11.32 mg·kg^−1^ for available phosphorus, and 106.23 mg·kg^−1^ for available potassium.

### 4.2. Experimental Design

**Figure 14:**
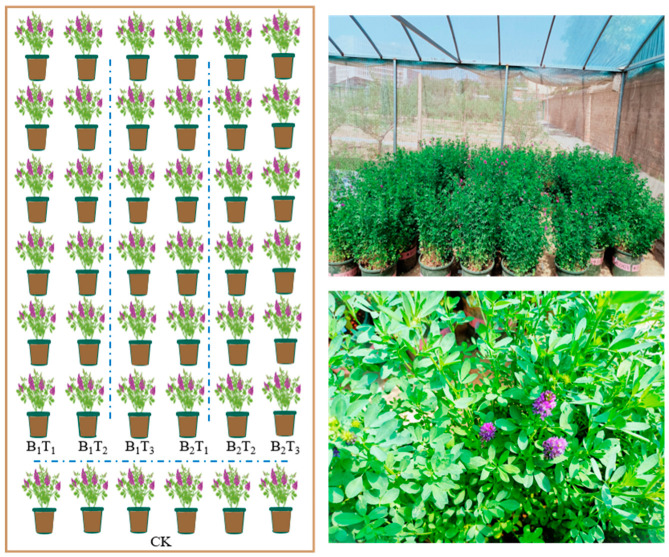
Distribution map of experimental design.

The alfalfa (*Medicago sativa* L.) variety used in this experiment was “Gannong No.3”, provided by the College of Grassland Science, Gansu Agricultural University. The experiment was conducted using pot cultivation, with a growing medium consisting of a uniform 5:1 mixture of experimental soil and potting soil. The experimental soil was collected from the forage practical training base of Gansu Agricultural University in Lanzhou, Gansu Province, and was of the loessial soil type. The nutrient soil was supplied by Shenxian Luyuan Seedling Substrate Co., Ltd. (Liaocheng, China) Prior to sowing, the growing substrate was placed into plastic pots with a rim diameter of 26 cm and a height of 31 cm, with a seeding rate of 40 seeds per pot. Two types of water-retaining agents were selected for the experiment: a starch-grafted acrylate water-retaining agent (B1, purchased from Renqiu Jinyu Chemical Co., Ltd. (Cangzhou, China)) and a polyacrylamide water-retaining agent (B2, purchased from Beijing Moore Chemical Technology Co., Ltd. (Beijing, China)). Four application levels were established: 0 kg·hm^−2^ (CK), 30 kg·hm^−2^ (T1), 60 kg·hm^−2^ (T2), and 90 kg·hm^−2^ (T3). The experiment comprised 7 treatments in total, each replicated 6 times, for a total of 42 pots (Figure 14). The soil in the lower one-third or more of each pot was thoroughly mixed with the corresponding amount of water-retaining agent. Alfalfa seeds were then evenly broadcast over the mixed soil layer, after which a layer of soil approximately 2 cm thick was applied and compacted, followed by thorough irrigation. The experiment was conducted under a rain shelter to enable precise soil moisture control. During the experimental period, the irrigation of the CK treatment (no water-retaining agent) was used as the benchmark. The soil moisture of the CK treatment was maintained at 65–75% of field capacity, and the irrigation amount per application for CK was calculated accordingly. All other treatments were irrigated with the same calculated amount of water. Irrigation was applied manually, and soil water content in the 0–20 cm soil layer was measured before and after each irrigation event using a PICO-BTTDR instrument (Ettlingen, Germany) to determine whether soil moisture had fallen below the lower irrigation threshold and to establish the timing and volume of irrigation. No fertilizer was applied throughout the entire growing period, alfalfa was allowed to grow entirely under natural conditions, and weeds were removed once every two weeks. Each cut was made at the early flowering stage. The first cut was performed on 26 June 2022 and 17 May 2023; the second cut on 31 July 2022 and 18 June 2023; the third cut on 6 September 2022 and 20 July 2023; and the fourth cut was not performed in 2022 (as 2022 was the establishment year, temperatures were relatively low during the growth period of the fourth cut, and it was necessary to ensure that the plants successfully overwintered, and therefore, the fourth cut was not harvested), while in 2023, the fourth cut was carried out on 25 August.

### 4.3. Sample Collection and Measurements

#### 4.3.1. Growth Indicators

Three pots with uniformly growing alfalfa plants were selected from each treatment and labeled with marker tags. Plant height (cm) was measured as the absolute height from the base to the apex of the plant using a steel tape measure. Stem diameter (mm) was measured at 5 cm above the soil surface inside the pot using a digital caliper (Harbin, China). The number of primary branches at the root crown was counted and averaged to obtain the primary branch number. The third fully expanded healthy intact leaf, counting downward from the apical leaf of each alfalfa plant, was selected, with 6 fresh leaves collected per pot, and leaf area (cm^2^) was determined using an LA-S leaf area scanner (Hangzhou, China).

#### 4.3.2. Physiological Indicators

Three plants were randomly selected from the labeled pot-grown alfalfa in each treatment. The third-to-fifth complete fresh leaves were clipped from the apex downward, immediately placed into aluminum foil bags, and flash-frozen in liquid nitrogen, then transported to the laboratory and stored in an ultra-low-temperature freezer at −80 °C for determination of the following indicators: soluble protein (SP) content (determined by Coomassie Brilliant Blue staining [43]), soluble sugar (SS) content (determined by the anthrone method [40]), proline (Pro) content (determined by the ninhydrin method [44]), superoxide dismutase (SOD), peroxidase (POD), and catalase (CAT) activities (all determined using assay kits provided by Suzhou Gracious Biotechnology Co., Ltd., Suzhou, China), and malondialdehyde (MDA) content (determined by the thiobarbituric acid method [45]).

#### 4.3.3. Yield

The labeled pot-grown alfalfa was cut at 5 cm above the soil surface and weighed to obtain fresh weight. The harvested fresh samples were placed in an oven, fixed at 105 °C for 30 min, and then dried at 75 °C for 48 h until a constant weight was reached. After the samples were cooled to room temperature, dry weight (g·pot^−1^) was recorded and converted to yield per hectare (Y, kg·hm^−2^).

#### 4.3.4. Water-Use Efficiency

(1) Water consumption (ET, mm) was calculated using the water balance method:

(1) $$\mathrm{ET}\;=\;\sum_{i=1}^{n}\gamma_{i}H_{i}\left(\theta_{i1}\;-{\;\theta }_{i2}\right)\;+\;I\;+\;P\;+\;K\;-\;R\;-\;D$$

i-soil layer number; *n*—total number of soil layers; $\gamma_{i}$-dry bulk density of the i-th soil layer (g·cm^−3^); H_i_-thickness of the i-th soil layer (cm); θ_i1_ and θ_i2_-soil water content (%) of the i-th layer at the beginning and end of the time interval, respectively, expressed as a percentage of dry soil mass; I-irrigation amount (mm); P-precipitation (mm); K-groundwater recharge (mm); R-surface runoff (mm); and D-deep percolation (mm). As this study employed a pot cultivation approach with a rain shelter used to exclude rainfall, P, K, R, and D were all neglected.

(2) Water-use efficiency (WUE, kg·m^−3^):

(2) $$\mathrm{WUE}\;=\;\frac{Y}{10\mathrm{ET}}$$

#### 4.3.5. Data Processing and Statistical Analysis

Experimental data were organized and calculated in Microsoft Excel 2016. IBM SPSS Statistics 26 software was used for statistical analysis. One-way analysis of variance (one-way ANOVA) and Duncan’s multiple range test were applied to compare plant height, stem diameter, leaf area, branch number, osmotic adjustment substances, antioxidant system parameters, yield, and WUE among different treatments (*p* < 0.05). Two-way analysis of variance (two-way ANOVA) was employed to examine the effects of water-retaining agent type, application rate, and their interaction (*p* < 0.05). During statistical analysis, the data from multiple cuts and years were not treated as a continuous time series. Treatments within each cut and each year of alfalfa were compared independently. This meant that differences among different treatments at the same time point were analyzed. Origin 2021 software was used for plotting, correlation analysis, and principal component analysis, wherein the correlation analysis was based on the average values of each indicator after multiple measurements. Before performing principal component analysis, all raw data were standardized using Z-score normalization to eliminate the influence of different units of measurement (e.g., cm, mg·g^−1^, U·g^−1^, etc.).

## 5. Conclusions and Perspectives

(1) The application of an appropriate amount of water-retaining agent can promote alfalfa growth and development. Under the same water-retaining agent type, alfalfa plant height, stem diameter, leaf area, and branch number generally first increased and then decreased with increasing application rate. Under the same water-retaining agent application level, alfalfa plant height, stem diameter, leaf area, and branch number generally followed the order B1 < B2.

(2) The application of an appropriate amount of water-retaining agent is conducive to increasing osmotic adjustment substance contents and antioxidant enzyme activities in alfalfa leaves, while reducing malondialdehyde (MDA) content. The B2T2 treatment yielded the highest SP content, SS content, and POD activity. The B1T2 treatment yielded the highest Pro content, SOD activity, and CAT activity, while the B2T3 treatment yielded the lowest MDA content.

(3) The application of an appropriate amount of water-retaining agent can significantly increase total alfalfa yield and water-use efficiency. Compared with CK, alfalfa yield and WUE under water-retaining agent application increased by 3.55~22.95% and 7.92~24.44%, respectively. Under the same water-retaining agent type, alfalfa yield and WUE followed the order T2 > T3 > T1. Under the same water-retaining agent application level, alfalfa yield and WUE followed the order B2 > B1.

Correlation analysis of alfalfa growth and physiological indicators, yield, and water-use efficiency revealed that yield was significantly positively correlated with plant height, leaf area, branch number, leaf SP content, leaf POD activity, and leaf CAT activity (*p* < 0.05). Comprehensive evaluation based on principal component analysis (PCA) indicated that T2 (60 kg·hm^−2^) combined with B2 (polyacrylamide water-retaining agent) achieved a high comprehensive score, and the B2T2 treatment had relatively high yield and WUE over the two years, preliminarily indicating that this combination can provide a theoretical basis and practical reference for the rational application of water-retaining agents in alfalfa production.

## Data Availability

The original contributions presented in this study are included in the article. Further inquiries can be directed to the corresponding authors.
